# Short-term prognosis of emergently hospitalized dialysis-independent chronic kidney disease patients: A nationwide retrospective cohort study in Japan

**DOI:** 10.1371/journal.pone.0208258

**Published:** 2018-11-29

**Authors:** Hiroaki Kikuchi, Eiichiro Kanda, Takayasu Mori, Hidehiko Sato, Soichiro Iimori, Naohiro Nomura, Shotaro Naito, Eisei Sohara, Tomokazu Okado, Shinichi Uchida, Kiyohide Fushimi, Tatemitsu Rai

**Affiliations:** 1 Department of Nephrology, Graduate School of Medicine and Dental Sciences, Tokyo Medical and Dental University, Tokyo, Japan; 2 Medical Science, Kawasaki Medical School, Okayama, Japan; 3 Department of Health Policy and Informatics, Graduate School of Medicine and Dental Sciences, Tokyo Medical and Dental University, Tokyo, Japan; International University of Health and Welfare, School of Medicine, JAPAN

## Abstract

In patients with chronic kidney disease (CKD), low body mass index (BMI) is associated with high mortality. This relationship in emergently hospitalized CKD patients is unknown. We investigated the association between obesity and short-term mortality in emergently admitted patients with dialysis-independent CKD (DI-CKD) with and without infection. This retrospective cohort study examined Diagnosis Procedure Combination data of 26103 emergently hospitalized DI-CKD patients. Patients were divided into 8 groups according to their BMI and the presence of infectious diseases. The primary outcome was in-hospital death within 100 days. Cox proportional hazards models adjusted for baseline characteristics showed that low BMI was associated with the outcome both in infected and in non-infected patients (reference group as non-infected and medium BMI [24–26 kg/m^2^] group): infected and the lowest BMI (≤20 kg/m^2^) group, hazard ratio (HR) 1.82 (95% confidence interval 1.51, 2.19); non-infected and the lowest BMI group, 1.39 (1.16, 1.67). When patients were stratified according to presence of diabetes mellitus (DM), patients with DM showed that low BMI was associated with the outcome both in infected and in non-infected patients, whereas in non-DM patients, this relationship was attenuated in the non-infected group. For emergently hospitalized CKD patients with infection, high BMI was associated with lower mortality irrespective of the DM status. For non-infected patients, the effects of obesity for in-hospital mortality were modified by the DM status.

## Introduction

Chronic kidney disease (CKD) is associated with an increased risk of cardiovascular diseases and mortality [1]. Although obesity is associated with higher mortality in the general population [2], this association is reversed in patients on dialysis [3,4], an effect called the “obesity paradox.” The reversal of the obesity–mortality association has been relatively consistent in dialysis patients, but there are limited studies showing conflicting results in dialysis-independent CKD (DI-CKD) patients [3,5,6]. Although the exact mechanism underlying the obesity paradox is unclear, it is thought that a well-preserved energy store may have a protective effect for the energy wasting condition induced by uremic toxins and other cytokines in CKD [7]. Thus, it is plausible that obesity is beneficial for patients with long-term wasting conditions. However, it was recently suggested that the obesity paradox results from flawed methodologies, such as population selection bias, lack of control for the severity of associated diseases, and time discrepancies among competing risk factors [8]. Given that patients with a high body mass index (BMI) might have more beneficial residual factors for survival than those with a lower BMI who are not included in the analyses, cohort studies with long-term follow-up may not be suitable for proving the “true” obesity paradox. Thus, in order to demonstrate an unbiased association between obesity and survival, it should be demonstrated that obesity is also beneficial for short-term prognosis, such as in-hospital mortality, of CKD patients under morbid conditions. However, to the best of our knowledge, no study has shown the obesity paradox of DI-CKD patients who are emergently admitted and are under highly stressed conditions.

Inflammation is one of the major causes for energy wasting and losing body weight and is closely associated with higher mortality and CKD progression in CKD patients [9–12]. A recent study showed that, in fully adjusted time-dependent analyses, higher BMI quintiles were not associated with a protective effect in non-inflamed patients, whereas higher BMI was associated with a lower all-cause mortality risk in inflamed patients on hemodialysis [11]. However, to the best of our knowledge, no study has investigated the interactive effects of inflammation on short-term protective effects of obesity in DI-CKD patients. Furthermore, it is also unknown which subgroups of non-inflamed obese CKD patients are associated with lower mortality. In this study, we investigated the short-term protective effects of obesity in emergently admitted DI-CKD patients with and without infectious disease.

## Results

### Comparison of baseline population characteristics and outcomes between groups

The mean age of the cohort was 59.2 ± 10.2 years, 72.2% (*n* = 19117) were males. The mean BMI was 23.9 ± 5.1 kg/m^2^. The baseline characteristics of the patients categorized by their baseline BMI are presented in Table 1. Patients with a higher BMI were younger; were more likely to be males; were less likely to be transported by ambulance; were more likely to be treated by vasopressors; were less likely to have received a blood transfusion; were less likely to have received a central venous catheter insertion; had a higher prevalence of diabetes mellitus (DM), hypertension; and had a lower prevalence of anemia. In terms of reason for admission, patients with a lower BMI were more likely to be admitted by hematemesis or hemoptysis and by consciousness disorder. Patients with a higher BMI were more likely to be admitted by severe respiratory failure or heart failure and by severe metabolic disorders. In-hospital mortality was higher in patients with a lower BMI (Table 1).

**Table 1:** Comparison of baseline population characteristics between groups.

Number	Whole Groups	Q1	Q2	Q3	Q4	*P*
	(n = 26103)	(n = 6529)	(n = 7068)	(n = 6078)	(n = 6428)	
***Patients demographics***						
BMI, kg/m^2^	59.2 ± 10.2	18.2 ± 1.8	22.0 ± 0.8	24.9 ± 0.8	30.6 ± 4.3	<0.001
Age, years	23.9 ± 5.1	59.6 ± 10.7	60.5 ± 9.5	60.0 ± 9.3	56.4 ± 10.7	<0.001
Male, n (%)	19117 (73.2)	4121 (63.1)	5355 (75.8)	4866 (80.1)	4775 (74.3)	<0.001
Ambulance transportation, n (%)	10655 (40.8)	2801 (42.9)	2950 (41.7)	2488 (40.9)	2416 (37.6)	<0.001
ICU admission, n (%)	2590 (9.9)	579 (8.9)	706 (10.0)	639 (10.5)	666 (10.4)	0.008
Vasopressor usage, n (%)	6657 (25.5)	1502 (23.0)	1767 (25.0)	1600 (26.3)	1788 (27.8)	<0.001
Blood transfusion, n (%)	3307 (12.7)	1140 (17.5)	930 (13.2)	669 (11.0)	568 (8.8)	<0.001
Central venous line usage, n (%)	3124 (12.0)	836 (12.8)	910 (12.9)	676 (11.1)	702 (10.9)	<0.001
***Comorbidities***						
Diabetes mellitus, n (%)	9096 (34.8)	1566 (24.0)	2288 (32.4)	2268 (37.3)	2974 (46.3)	<0.001
Hypertension, n (%)	13370 (51.2)	2464 (37.7)	3517 (49.8)	3422 (56.3)	3967 (61.7)	<0.001
Anemia, n (%)	2047 (7.8)	666 (10.2)	548 (7.8)	438 (7.2)	395 (6.1)	<0.001
Infectious disease, n (%)	6993 (26.8)	2113 (32.4)	1911 (27.0)	1463 (24.1)	1506 (23.4)	<0.001
Malignancy, n (%)	3211 (12.3)	1065 (16.3)	916 (13.0)	677 (11.1)	553 (8.6)	<0.001
***Reason for admission***						
Hematemesis or hemoptysis, n (%)	2057 (7.9)	722 (11.1)	558 (7.9)	417 (6.9)	360 (5.6)	<0.001
Consciousness disorder, n (%)	2338 (9.0)	663 (10.2)	715 (10.1)	519 (8.5)	441 (6.9)	<0.001
Severe respiratory failure or Heart failure, n (%)	6290 (24.1)	1187 (18.2)	1553 (22.0)	1549 (25.5)	2001 (31.1)	<0.001
Severe metabolic disorder, n (%)	5662 (21.7)	1431 (21.9)	1517 (21.5)	1273 (20.9)	1441 (22.4)	<0.001
Emergent operation or catheter intervension or t-PA treatment	2773 (10.6)	631 (9.7)	778 (11.0)	682 (11.2)	682 (10.6)	0.371
***Outcome***						
In-hospital death, n (%)	1729 (6.6)	656 (10.0)	486 (6.9)	325 (5.3)	262 (4.1)	<0.001
Duration of hospital stay, days	23.0 ± 20.6	25.0 ± 23.2	22.3 ± 19.9	21.9 ± 19.2	22.7 ± 19.5	<0.001

The values of normally distributed variables are presented as the mean ± standard deviation (SD). Categorical data are presented as values and percentages. BMI, body mass index; ICU, intensive care unit; t-PA, tissue plasminogen activator.

### The lowest BMI groups had the highest in-hospital mortality rates irrespective of the inflammation status in emergently admitted DI-CKD patients

**Fig 1:**
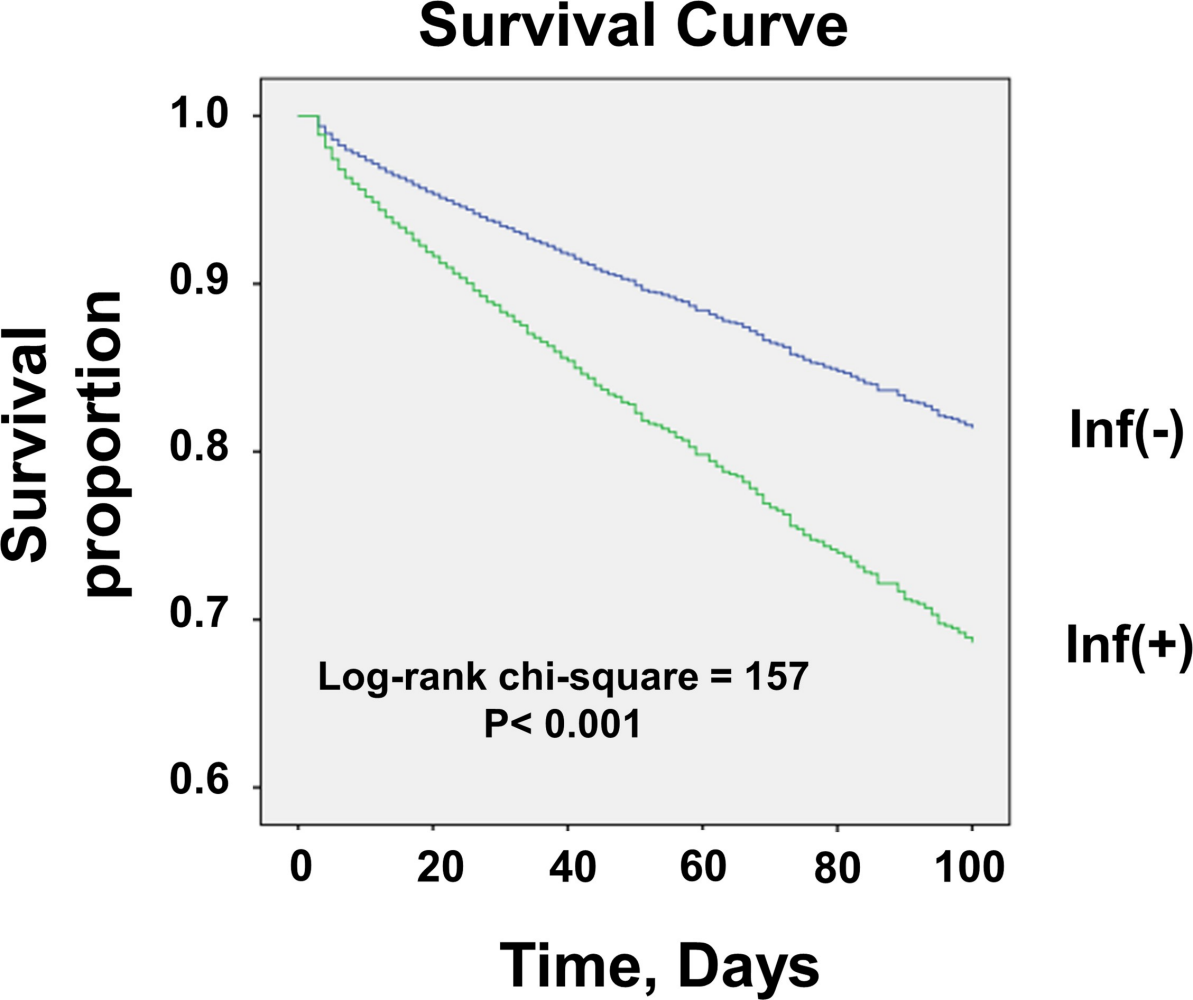
Kaplan–Meier estimates of all-cause mortality for emergently admitted DI-CKD patients stratified by infection status. Infected DI-CKD patients had a significantly lower survival rate than non-infected DI-CKD patients (*P* < 0.001). Inf: infection.

**Fig 2:**
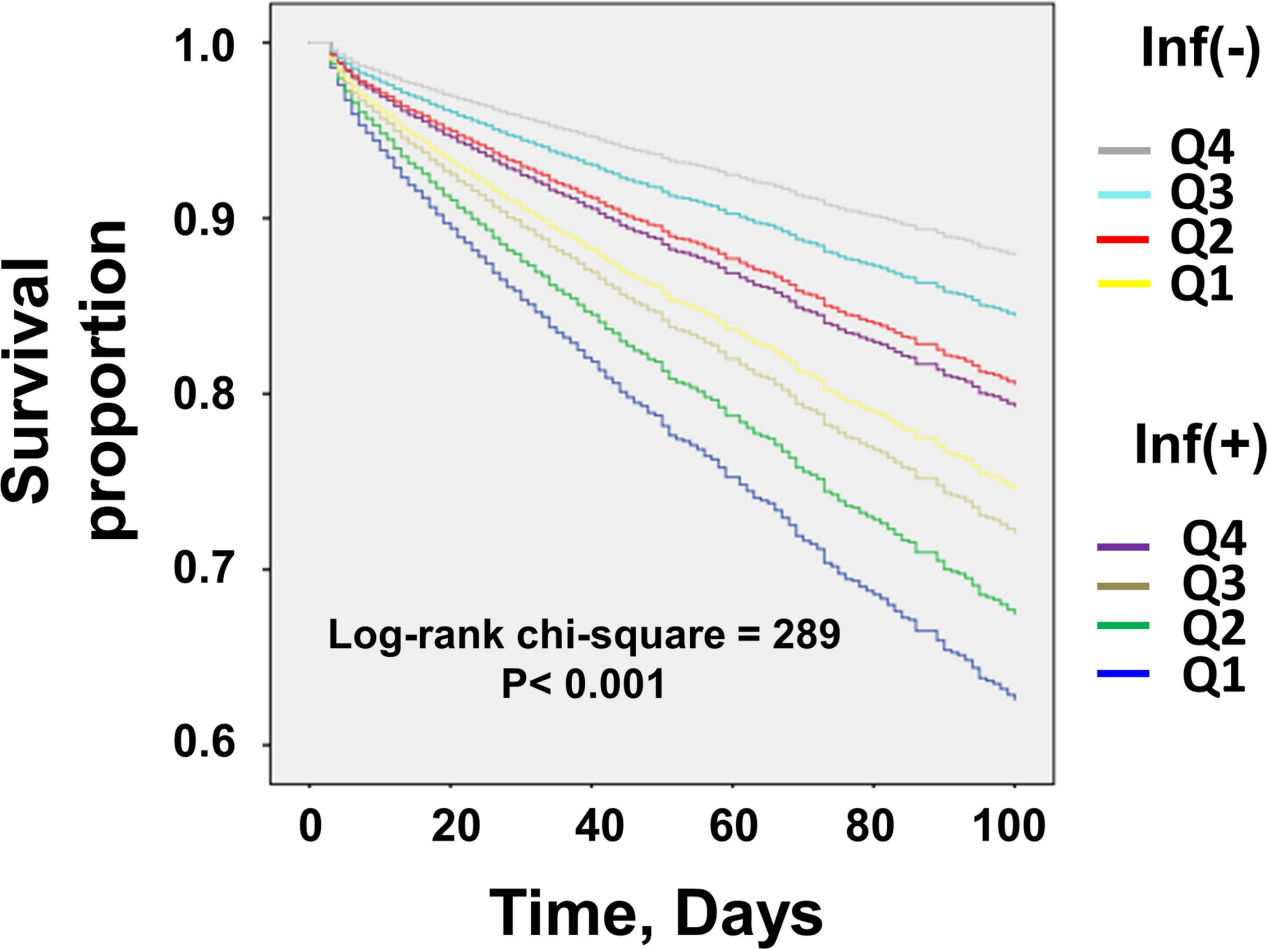
Kaplan–Meier estimates of all-cause mortality for emergently admitted DI-CKD patients stratified by infection status and BMI quartiles. The lowest BMI quartile (Q1) has the highest in-hospital mortality rate irrespective of the infection status in emergently admitted DI-CKD patients. The highest BMI quartile (Q4) shows a survival advantage in both infected and non-infected DI-CKD patients. CKD: chronic kidney disease; Inf: infection.

We hypothesized that the relationship between BMI and short-term mortality in emergently admitted DI-CKD patients was influenced by infectious diseases. Thus, we stratified DI-CKD patients by the presence or absence of infectious diseases. From the ICD-10 revision code (2003 version), we included pulmonary infection, gastrointestinal infection, genitourinary infection, soft tissue infection, and septicemia as infectious disease. (S1 Table) In a Kaplan–Meier analysis, infected DI-CKD patients had a statistically significant lower survival rate than non-infected DI-CKD patients (*P* < 0.001) (Fig 1). The lowest BMI quartile (Q1) had the highest mortality both in infected and in non-infected DI-CKD patients (Fig 2, Table 2). The highest BMI quartile (Q4) showed survival advantages in both infected and non-infected DI-CKD patients. However, in non-infected patients, the difference in the hazard ratios (HRs) among the highest quartile (Q4), the second-highest quartile (Q3) and the second-lowest quartile (Q2) were not statistically significant (Table 2). Subsequently, we scrutinized the populations in which obesity is less or more favorable for short-term survival in DI-CKD patients.

**Table 2:** All-cause mortality by BMI and infection in DI-CKD patients; Cox proportional hazards analysis.

Model	BMI quartile	Infection Present			Infection Absent		
		HR (95% CI)		*P*	HR (95% CI)		*P*
I	Q1 (≤20 kg/m2)	2.77	(2.31, 3.33)	<0.001	1.74	(1.45, 2.08)	<0.001
	Q2 (21–23 kg/m2)	2.33	(1.92, 2.84)	<0.001	1.28	(1.06, 1.55)	0.010
	Q3 (24–26 kg/m2)	1.94	(1.56, 2.41)	<0.001	1	Ref	
	Q4 (≥27 kg/m2)	1.37	(1.08, 1.75)	0.009	0.76	(0.62, 0.95)	0.014
II	Q1 (≤20 kg/m2)	1.92	(1.59, 2.31)	<0.001	1.40	(1.17, 1.68)	<0.001
	Q2 (21–23 kg/m2)	1.69	(1.38, 2.05)	<0.001	1.19	(0.99, 1.44)	0.069
	Q3 (24–26 kg/m2)	1.56	(1.25, 1.94)	<0.001	1	Ref	
	Q4 (≥27 kg/m2)	1.26	(0.99, 1.60)	0.063	0.91	(0.74, 1.13)	0.397
III	Q1 (≤20 kg/m2)	1.82	(1.51, 2.19)	<0.001	1.39	(1.16, 1.67)	<0.001
	Q2 (21–23 kg/m2)	1.44	(1.18, 1.75)	<0.001	1.16	(0.96, 1.40)	0.124
	Q3 (24–26 kg/m2)	1.40	(1.12, 1.74)	0.003	1	Ref	
	Q4 (≥27 kg/m2)	1.06	(0.84, 1.35)	0.623	0.92	(0.74, 1.14)	0.426

Model I: non-adjusted. Model II: adjusted for demographics, medical history: age, sex, history of DM, hypertension, anemia, and malignancy. Model III adjusted for all variables in model II plus reason for admission, history of ambulance transportation, history of ICU admission, history of vasopressor usage, history of blood transfusion, and history of usage of central venous line.

HR, hazard ratio; CI, confidence interval; BMI, body mass index; Ref, reference

### DM modifies the paradoxical association between BMI and all-cause mortality in non-infected emergently admitted DI-CKD patients

**Fig 3:**
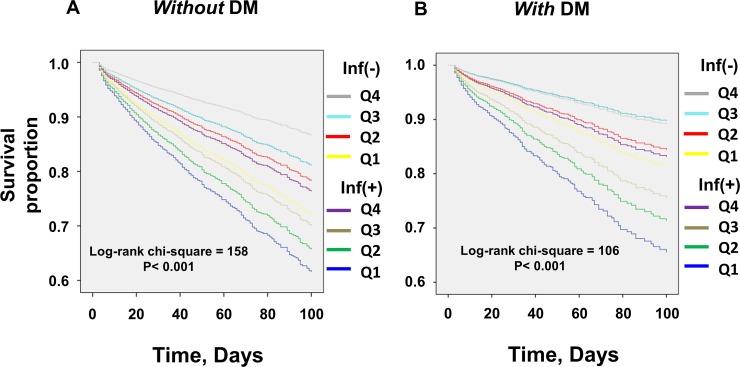
Unadjusted Kaplan–Meier estimates of all-cause mortality for DI-CKD patients stratified by complication with the DM status. (A) Kaplan–Meier estimates of all-cause mortality for DI-CKD patients *without* DM. (B) Kaplan–Meier estimates of all-cause mortality for DI-CKD patients *with* DM. In non-infected DI-CKD patients, the association of higher BMI with mortality was more evident in DM patients, whereas in infected DI-CKD patients, obesity had a favorable association with mortality irrespective of the complication of DM. DM: diabetes mellitus; CKD: chronic kidney disease; Inf: infection.

We stratified the participants into DM and non-DM groups and investigated the relationship between BMI and all-cause mortality for the DM and non-DM DI-CKD patients. In the non-DM patients, Kaplan–Meier estimates suggested a survival advantage for the highest BMI categories both in infected and in non-infected patients (Fig 3A). However, in an adjusted model, Cox proportional hazards model analysis showed that there were no statistically significant differences among Q2, Q3, and Q4 in non-DM non-infected patients (Table 3). On the other hand, in DM non-infected patients, Kaplan–Meier estimates showed that patients in the second-highest BMI quartile (Q3) had survival advantages compared with the other BMI quartiles (Fig 3B). Cox proportional hazards model analysis also showed that, in non-infected patients, the second-highest BMI quartile (Q3) had statistically significant survival advantages compared with the second-lowest BMI quartile (Q2) (Table 4) in contrast to the results for the non-DM patients. To scrutinize these results found in non-infected patients, we further stratified patients by whether they were admitted for “severe respiratory failure or heart failure” or not. As shown in Table 5, in non-infected DM patients, the paradoxical association was more evident in the patients admitted for the reasons “other than severe respiratory failure or heart failure”. On the other hand, in the non-infected non-DM patients, this paradoxical association was more evident in patients admitted for “severe respiratory failure or heart failure” (Table 5).

**Table 3:** All-cause mortality by BMI and infection in DI-CKD patients without DM; Cox proportional hazards analysis.

Model	BMI quartile	Infection Present			Infection Absent		
		HR (95% CI)		*P*	HR (95% CI)		*P*
I	Q1 (≤20 kg/m^2^)	2.32	(1.88, 2.86)	<0.001	1.57	(1.28, 1.93)	<0.001
	Q2 (21–23 kg/m^2^)	2.01	(1.60, 2.52)	<0.001	1.17	(0.94, 1.46)	0.165
	Q3 (24–26 kg/m^2^)	1.70	(1.31, 2.21)	<0.001	1	Ref	
	Q4 (≥27 kg/m^2^)	1.29	(0.96, 1.72)	0.087	0.68	(0.52, 0.90)	0.006
II	Q1 (≤20 kg/m^2^)	1.71	(1.39, 2.12)	<0.001	1.31	(1.06, 1.61)	0.011
	Q2 (21–23 kg/m^2^)	1.53	(1.21, 1.92)	<0.001	1.07	(0.86, 1.33)	0.544
	Q3 (24–26 kg/m^2^)	1.39	(1.07, 1.81)	0.013	1	Ref	
	Q4 (≥27 kg/m^2^)	1.19	(0.89, 1.60)	0.234	0.81	(0.61, 1.06)	0.116
III	Q1 (≤20 kg/m^2^)	1.62	(1.31, 2.00)	<0.001	1.34	(1.09, 1.66)	0.006
	Q2 (21–23 kg/m^2^)	1.30	(1.03, 1.63)	0.027	1.12	(0.90, 1.40)	0.319
	Q3 (24–26 kg/m^2^)	1.20	(0.93, 1.57)	0.167	1	Ref	
	Q4 (≥27 kg/m^2^)	0.93	(0.70, 1.25)	0.632	0.87	(0.66, 1.14)	0.299

Model I: non-adjusted. Model II: adjusted for demographics, medical history: age, sex, history of DM, hypertension, anemia, and malignancy. Model III adjusted for all variables in model II plus reason for admission, history of ambulance transportation, history of ICU admission, history of vasopressor usage, history of blood transfusion, and history of usage of central venous line.

HR, hazard ratio; CI, confidence interval; BMI, body mass index; Ref, reference

**Table 4:** All-cause mortality by BMI and infection in DI-CKD patients with DM; Cox proportional hazards analysis.

Model	BMI quartile	Infection Present			Infection Absent		
		HR (95% CI)		*P*	HR (95% CI)		*P*
I	Q1 (≤20 kg/m^2^)	3.89	(2.68, 5.64)	<0.001	1.91	(1.31, 2.78)	<0.001
	Q2 (21–23 kg/m^2^)	3.12	(2.12, 4.58)	<0.001	1.56	(1.09, 2.25)	0.01
	Q3 (24–26 kg/m^2^)	2.58	(1.71, 3.89)	<0.001	1	Ref	
	Q4 (≥27 kg/m^2^)	1.71	(1.11, 2.62)	0.002	1.06	(0.73, 1.54)	0.811
II	Q1 (≤20 kg/m^2^)	3.14	(2.16, 4.56)	<0.001	1.73	(1.18, 2.52)	0.005
	Q2 (21–23 kg/m^2^)	2.65	(1.80, 3.90)	<0.001	1.52	(1.06, 2.18)	0.024
	Q3 (24–26 kg/m^2^)	2.28	(1.51, 3.44)	<0.001	1	Ref	
	Q4 (≥27 kg/m^2^)	1.54	(1.06, 2.51)	0.026	1.16	(1.00, 1.03)	0.446
III	Q1 (≤20 kg/m^2^)	2.80	(1.92, 4.08)	<0.001	1.59	(1.09, 2.32)	0.016
	Q2 (21–23 kg/m^2^)	1.95	(1.32, 2.89)	<0.001	1.40	(0.98, 2.01)	0.061
	Q3 (24–26 kg/m^2^)	1.90	(1.26, 2.87)	0.003	1	Ref	
	Q4 (≥27 kg/m^2^)	1.17	(0.76, 1.81)	0.444	1.13	(0.77, 1.64)	0.532

Model I: non-adjusted. Model II: adjusted for demographics, medical history: age, sex, history of DM, hypertension, anemia, and malignancy. Model III adjusted for all variables in model II plus reason for admission, history of ambulance transportation, history of ICU admission, history of vasopressor usage, history of blood transfusion, and history of usage of central venous line.

HR, hazard ratio; CI, confidence interval; BMI, body mass index; Ref, reference

**Table 5:** Reason for admission modifies all-cause mortality by BMI in non-infected DI-CKD patients; Cox proportional hazards analysis.

		Reason for Admission					
		Severe respiratory failure or heart failure			Other than severe respiratory failure or heart failure		
	**BMI quartile**	**HR (95% CI)**		***P***	**HR (95% CI)**		***P***
**All**	Q1 (≤20 kg/m^2^)	1.58	(1.05, 2.38)	0.027	1.32	(1.07, 1.62)	0.008
	Q2 (21–23 kg/m^2^)	1.33	(0.87, 2.03)	0.184	1.10	(0.89, 1.36)	0.360
	Q3 (24–26 kg/m^2^)	1	Ref		1	Ref	
	Q4 (≥27 kg/m^2^)	0.73	(0.45, 1.19)	0.209	1.02	(0.80, 1.30)	0.867
	**BMI quartile**	**HR (95% CI)**		***P***	**HR (95% CI)**		***P***
***Without* DM**	Q1 (≤20 kg/m^2^)	1.66	(1.01, 1.04)	0.048	1.20	(0.95, 1.51)	0.125
	Q2 (21–23 kg/m^2^)	1.57	(0.93, 2.66)	0.093	0.98	(0.77, 1.24)	0.838
	Q3 (24–26 kg/m^2^)	1	Ref		1	Ref	
	Q4 (≥27 kg/m^2^)	0.63	(0.31, 1.26)	0.190	0.95	(0.71, 1.27)	0.709
	**BMI quartile**	**HR (95% CI)**		***P***	**HR (95% CI)**		***P***
***With* DM**	Q1 (≤20 kg/m^2^)	1.34	(0.63, 2.83)	0.446	1.68	(1.08, 2.61)	0.023
	Q2 (21–23 kg/m^2^)	1.03	(0.51, 2.10)	0.926	1.59	(1.03, 2.44)	0.035
	Q3 (24–26 kg/m^2^)	1	Ref		1	Ref	
	Q4 (≥27 kg/m^2^)	0.79	(0.39, 1.57)	0.502	1.33	(0.85, 2.07)	0.211

Model: adjusted for demographics, medical history: age, sex, history of DM, hypertension, anemia, and malignancy, plus reason for admission, history of ambulance transportation, history of ICU admission, history of vasopressor usage, history of blood transfusion, and history of usage of central venous line.

HR, hazard ratio; CI, confidence interval; BMI, body mass index; Ref, reference

## Discussion

In our study of emergently admitted DI-CKD patients, we demonstrated that the paradoxical association between higher BMI and better in-hospital mortality was seen both in infected and in non-infected patients, although this association was relatively weaker in the non-infected patients. Surprisingly, in the non-infected patients, this paradoxical association was strengthened in patients with DM. To the best of our knowledge, this is the first study demonstrating the favorable association between obesity and short-term prognosis in emergently admitted DI-CKD patients.

Conventional BMI classifications are derived primarily in European populations to correspond to risk thresholds for a wide range of chronic diseases and mortality [13]. However, there is an ongoing debate on whether these criteria are appropriate for non-European populations. For example, there was increasing evidence of the emerging high prevalence of type 2 diabetes and increased cardiovascular risk factors in parts of Asia where the average BMI is below the cutoff point of 25 kg/m^2^ that defines overweight in the current World Health Organization (WHO) classification [14]. Thus, for the evaluation of the obesity paradox in an Asian population, a quartile classification may be more suitable than the WHO classification.

Presently, the protective effects of obesity for short-term prognosis remain controversial. In a previous meta-analysis, obesity and morbid obesity were associated with lower mortality in patients with acute respiratory distress syndrome [15]. Another cohort study of surgical intensive care unit patients demonstrated that being overweight or obese was associated with a decreased risk of 60-day in-hospital mortality [16]. A recent study also showed that obesity was independently associated with reduced in-hospital mortality in patients with severe soft tissue infection [17]. On the other hand, one study showed that obese patients had higher all-cause mortality at 30 days after surgical, endoscopic, or obstetrical procedures than those that are not obese [18]. Another study showed that obesity was independently associated with higher in-hospital mortality in patients undergoing myocardial infarction [19]. Although the reasons for admission varied in our study, we demonstrated in this large cohort study that obesity exerted its protective effects on in-hospital mortality for both infected and non-infected DI-CKD patients.

Inflammation is a major risk factor for wasting of nutritional status, and a recent study showed that the obesity paradox was modified by the inflammation status in dialysis-dependent CKD patients [10,11]. Consistent with these studies, we found that the favorable association between obesity and short-term prognosis was more evident in infected patients than in non-infected patients (Table 2). Nonetheless, we found that obesity also had a favorable association with short-term mortality in non-infected CKD patients (Table 2), which has not been reported in previous studies. This discrepancy may first be attributable to the difference in the patient status at the study’s start point. In our study, emergently admitted CKD patients were likely to be suffering from severely stressed conditions. Therefore, some patients categorized as “non-infected” may also have experienced complications with comorbid wasting conditions, such as congestive heart failure and cerebrovascular events [20]. The second explanation for this is the difference in the study follow-up periods. Obesity is known to have deleterious effects as a long-term killer rather than as a short-term killer [21]. Thus, it is possible that the favorable role of obesity was more likely to be evident in a short-term follow-up study.

In our study, it is also noteworthy that the favorable association of obesity with in-hospital mortality was more evident in DM patients than in non-DM patients without infection (Tables 3 and 4). Even when the primary outcome of this study was changed from 100 day mortality to 60 day mortality, this effect was consistently evident (S2, S3 and S4 Tables). Given that obesity accounts for many of the risks for type 2 diabetes [22], our result was surprising. Thus, we further scrutinized this paradoxical association in non-infected patients by stratifying the patients whether they are admitted for “severe respiratory failure or heart failure” or not. We found that in DM patients, this paradoxical association in non-infected DM patients was weaker in patients admitted for “severe respiratory failure or heart failure”. Some, but not all, studies have indicated that protein energy wasting is more prevalent in diabetic than in nondiabetic CKD patients [23,24]. Thus, it seems that the favorable role of obesity is partly dependent on the reason for admission in non-infected patients.

There are several limitations in this study. Some of the potential residual confounders, such as laboratory data, functional status, degree of social support, and adherence to medical treatment, were unavailable. Furthermore, results of this study may have been limited due to the lack of severity score such as Acute Physiology and Chronic Health Evaluation (APACHE) score [25]. In this study, in order to estimate the severity of participants, treatments such as vasopressor usage and blood transfusion, and procedures including central venous catheter usage, and demographics including ambulance transportation and ICU admission were used for covariates. However, predictive variables for prognosis such as vital signs on admission [26], PaO_2_ /FiO_2_ ratio [27], and Glasgow Coma Scale at the time of admission [28] were unavailable. Also, laboratory measures of inflammation, such as white blood cell count, C-reactive protein, and serum albumin, were unavailable in this DPC system. Thus, the possibility that the lower BMI group might have included more participants with severer status compared to the higher BMI group is not excluded.

Other limitations are as follows. First, laboratory measures of kidney functions, such as serum creatinine were not available in this DPC system. Thus, it is possible that the number of CKD patients could be underreported. Considering the character of the DPC system, CKD population with lower severity could be more likely to be underreported. Thus, generalizability of the results of our study to early stage CKD patients may be limited. Second, we could not precisely separate CKD stages using ICD-10 codes. Third, BMI is a crude marker of body composition and does not accurately differentiate between lean and fat tissue. Fourth, BMI was calculated at the time of the patients’ admission, thus, the effects of fluctuation of BMI during hospitalization could not be evaluated in this study. Fifth, our study was restricted to Japanese patients, and its generalizability to other races may be limited. Sixth, our study was restricted to emergently admitted patients, and its generalizability to electively admitted CKD patients may be limited. Seventh, in this DPC system, patients with admission periods from one academic year of Japan (finishing March 31th) to the next academic year (starting from April 1st) were followed only 3 month at maximum. As the primary outcome of this study was in-hospital death within 100 days, this effect would be negligible. Finally, due to the fact that this was an observational study, causality could not be inferred.

In conclusion, this is the first study showing that higher BMI might have favorable effects on in-hospital mortality of emergently admitted infected and non-infected DI-CKD patients. In non-infected DM patients, especially who admitted by reasons other than severe respiratory failure or heart failure, this favorable effect of higher BMI on short-term mortality was amplified. The strong point of this study was its large sample size using nation-wide DPC system. The weak point of this study was the limited information of patients’ characteristics due to the nature of the dataset of DPC. Future studies are required to determine which body components are beneficial for the survival of DI-CKD patients and whether weight gain has protective effects on DI-CKD patients.

## Materials and methods

### Study design and setting

This was a retrospective observational cohort study using data from nationwide administrative claims and a discharge abstract database in Japan, the DPC database. In brief, more than 1000 hospitals, including all 81 academic hospitals in Japan, contribute to the DPC database. The annual number of cases added to the database is nearly 7 000 000 and encompasses nearly 50% of all hospital admissions in Japan [29].

The DPC database contains the following information for each patient: patient demographics, a unique hospital identifier, diagnoses, outcomes, drugs used, procedures performed, healthcare costs, and several disease-specific data. Patient demographics include BMI, age, sex, the reasons of admission, ICU admission, and vasopressor usage. Dopamine, dobutamine, norepinephrine, epinephrine, isoproterenol, milrinone, and vasopressin were included as vasopressors. Diagnoses include the main diagnosis on admission and existing comorbidities, which are recorded separately with the International Classification of Diseases-10th (ICD-10) revision codes 2003 version and text data in Japanese. This study was approved by the Institutional Review Board of Tokyo Medical and Dental University (no. M2000-788) and conducted according to the principles of the Declaration of Helsinki. Informed consent was waived because of the anonymous nature of the data.

### Study participants

**Fig 4:**
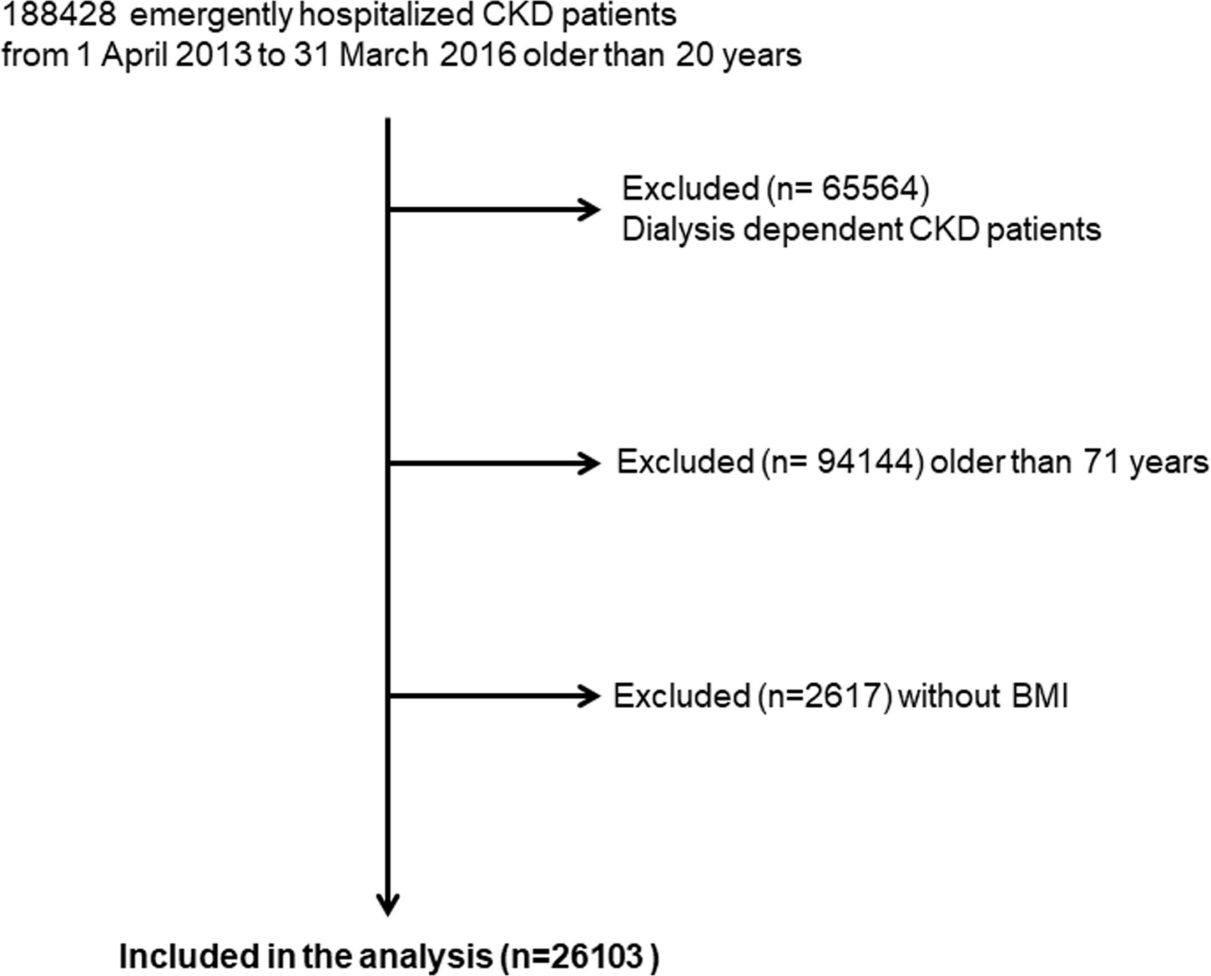
Flow chart for recruitment of participants and subjects studied. CKD: chronic kidney disease; BMI: body mass index.

Patients were eligible for inclusion in the present study if (1) they were emergently admitted to hospitals participating in the DPC system from April 2013 to March 2016, (2) they were above 20 years old and under 70 years old, (3) they had CKD independent of dialysis identified by the ICD-10 revision codes of N-289, N-180, N-188 and N-189, and (4) their BMI values on admission were recorded. Dialysis dependency, including peritoneal dialysis, and ICU admission, history of blood transfusion, history of central venous catheter insertion were identified by the receipt of healthcare cost of dialysis, ICU admission, blood transfusion, and catheter insertion during hospitalization in the DPC system, respectively. Furthermore, in order to exclude the dialysis dependent patients who did not receive dialysis treatment during hospitalization, patients who reached the outcome within 2 days were all excluded in this study. In light of the effects on BMI, patients who had undergone a lower extremity amputation were excluded. The present analysis included 26103 subjects (Fig 4). All patients’ data were followed up until they reached the outcome or until they were discharged from hospitals within 100 days after admission. When patients were emergently admitted to the same hospitals twice or more during the study period, only data from their first admission were used for subsequent analyses.

### Data collection and processing

Emergently admitted patients were identified as non-electively admitted patients with either of the following emergency medical cares on admission; (1) hematemesis and/or hemoptysis, (2) consciousness disorder and/or coma, (3) severe status of respiratory failure and/or heart failure, (4) acute medicinal poisoning, (5) shock vital, (6) severe metabolic disorder including liver failure, renal failure, or severe diabetes mellitus, (7) wide range heat burn, (8) severe trauma and/or tetanus, (9) severe status in which either emergent operation, emergent catheter intervention, or tissue plasminogen activator treatment was necessary, (10) other severe status which was comparable to above (1) to (9) status. Data on demographics, existing comorbidities, and several hospital characteristics were extracted for each patient. From the ICD-10 revision (2003 version) code, existing comorbidities included diabetes mellitus (E10-E14, including insulin dependent and non-dependent), hypertension (I10-I15, including primary and secondary hypertension, and hypertensive heart and renal diseases), anemia (D50-D59, including nutritional anemia and hemolytic anemia), infectious diseases and malignancies. Infectious diseases included pulmonary infection, gastrointestinal infection, genitourinary infection, soft tissue infection, and septicemia. BMI on admission, age and sex were also extracted from the DPC system. Patients with an ICD-10 code of N-289 or N-180 or N-188 or N-189 without receipt of hemodialysis were selected as dialysis-independent CKD patients. All ICD-10 codes used for this study are shown in S1 Table.

### Study groups and study outcome

Patients were divided into quartiles according to BMI: Quartile 1 (Q1), very low BMI (≤20 kg/m^2^); Quartile 2 (Q2), low BMI (21–23 kg/m^2^); Quartile 3 (Q3), high BMI (24–26 kg/m^2^); Quartile 4 (Q4), very high BMI (≥27 kg/m^2^). After this, patients were classified according to the presence (*n* = 6993) or absence (*n* = 19110) of infection as described above. Subsequently, we stratified the participants into DM and non-DM groups and investigated the relationship between BMI and all-cause mortality for DM and non-DM DI-CKD patients separately. Non-infected patients in BMI Q3 formed the reference group. The primary outcome was the occurrence of in-hospital death.

### Statistical analysis

The values of normally distributed variables are presented as the mean ± standard deviation (SD). Categorical data are presented as values and percentages. Intergroup comparisons were performed using the χ^2^ test and one-way analysis of variance, as appropriate. The primary analysis of interest was the relationship between BMI and all-cause mortality. Kaplan–Meier survival curves were constructed and log-rank testing was performed in order to assess the time to the incidence of the primary outcome. After the evaluation of proportional hazards by log minus log plots, a Cox proportional hazards model was used to evaluate the association between the BMI groups and the primary outcome. The multivariate Cox proportional hazards models were adjusted for the demographics, comorbidities and reasons for admission, namely, age, sex, history of ambulance transportation, history of ICU admission, history of vasopressor usage, comorbidities of DM, hypertension and anemia. In the multivariate analysis, age was used as categorical variables defined by quartiles. Among 10 categories of reasons for admission described above, (1), (2), (3), (6) and (9) were the top 5 major categories for the reason for admission. Thus, we included these 5 categories as variables for adjustment. The results are shown as HRs and 95% confidence intervals (CIs). Statistical analyses were performed using the Statistical Package for the Social Sciences (SPSS), version 20.0 (SPSS, Inc., Chicago, IL, USA). Statistical significance was defined as *P* < 0.05.

## Supporting information

S1 Table ICD-10^th^ codes (2003 version) for this study. (DOCX)

S2 Table Comparison of All-cause mortality by BMI and infection in DI-CKD patients, within 100 days, 60days and 30days. (DOCX)

S3 Table Comparison of All-cause mortality by BMI and infection in DI-CKD patients *without* DM, within 100 days, 60days and 30days. (DOCX)

S4 Table Comparison of All-cause mortality by BMI and infection in DI-CKD patients *with* DM, within 100 days, 60days and 30days. (DOCX)
