# Supplementary material for: Short-term prognosis of emergently hospitalized dialysis-independent chronic kidney disease patients: A nationwide retrospective cohort study in Japan
Source: PLoS One. 2018 Nov 29;13(11):e0208258. doi: 10.1371/journal.pone.0208258 (PMC6264841; doi:10.1371/journal.pone.0208258)
Supplement: S1 Table — (DOCX) [file pone.0208258.s001.docx]

**S1 Table. ICD-10^th^ codes (2003 version) for this study**

| ***Diseases*** | **ICD-10 codes (2013 version)** | | | | | | | | | |
| --- | --- | --- | --- | --- | --- | --- | --- | --- | --- | --- |
| **CKD** | N18.0 | N18.8 | N18.9 | N28.9 |  |  |  |  |  |  |
| **DM** | E10 | E11 | E12 | E13 | E14 |  |  |  |  |  |
| **HTN** | I10 | I11.X | I12.X | I13.X | I15.X |  |  |  |  |  |
| **Anemia** | D50.X | D51.X | D52.X | D53.X | D55.X | D56.X | D57.X | D58.X | D59.X |  |
| **Solid Malignancy** | C00.X | C01 | C02.X | C03.X | C04.X | C05.X | C06.X | C07.X | C08.X | C09.X |
|  | C10.X | C11.X | C12 | C13.X | C14.X | C15.X | C16.X | C17.X | C18.X | C19 |
|  | C20 | C21.X | C22.X | C23 | C24.X | C25.X | C26.X |  |  |  |
|  | C30.X | C31.X | C32.X | C33 | C34.X | C37 | C38.X | C39.X |  |  |
|  | C40.X | C41.X | C43.X | C44.X | C45.X | C46.X | C47.X | C48.X | C49.X |  |
|  | C50.X | C51.X | C52 | C53.X | C54.X | C55 | C56 | C57.X | C58 |  |
|  | C60.X | C61 | C62.X | C63.X | C64.X | C65 | C66 | C67.X | C68.X | C69.X |
|  | C70.X | C71.X | C72.X | C73.X | C74.X | C75.X |  |  |  |  |
|  | D00.X | D01.X | D02.X | D03.X | D04.X | D05.X | D06.X | D07.X | D09.X |  |
| **Non-solid malignancy** | C81.X | C82.X | C83.X | C84.X | C85.X | C88.X | C89 |  |  |  |
|  | C90.X | C91.X | C92.X | C93.X | C94.X | C95.X | C96.X |  |  |  |
| **Pulmonary infection** | A15.X | A16.X | A17.X | A18.X | A19.X |  |  |  |  |  |
|  | J12.X | J13 | J14 | J15.X | J16.X | J17.X | J18.X |  |  |  |
|  | J67.X | J68.X | J69.X |  |  |  |  |  |  |  |
| **GI infection** | A00.X | A01.X | A02.X | A03.X | A04.X | A05.X | A06.X | A07.X | A08.X | A09.X |
|  | K35.X | K36.X | K37.X | K38.X | K57.X | K63.0 | K63.1 |  |  |  |
|  | K65.X | K66.X | K67.X | K73.X | K75.0 |  |  |  |  |  |
|  | K80.X | K81.X | K82.X | K83.X | K85.X |  |  |  |  |  |
| **GU infection** | N10 | N11.X | N12 | N13.X |  |  |  |  |  |  |
|  | N30.X | N34.X | N39.0 | N41.X | N70.X |  |  |  |  |  |
| **sepsis** | A40.X | A41.X |  |  |  |  |  |  |  |  |
| **Soft　tissue infection** | L03.X | L08.X | A48.0 |  |  |  |  |  |  |  |
| **Other infection** | A49.X | G00.X | G03.X | G04.X | G06.X | G09 | I33.X | M00.X | M86.X |  |
| **Amputation (lower)** | S78.X | S98.X | T13.6 | Z89.5 | Z89.6 |  |  |  |  |  |
