# Supplementary material for: Short-term prognosis of emergently hospitalized dialysis-independent chronic kidney disease patients: A nationwide retrospective cohort study in Japan
Source: PLoS One. 2018 Nov 29;13(11):e0208258. doi: 10.1371/journal.pone.0208258 (PMC6264841; doi:10.1371/journal.pone.0208258)
Supplement: S2 Table — (DOCX) [file pone.0208258.s002.docx]

**S2 Table. Comparison of All-cause mortality by BMI and infection in DI-CKD patients, within 100 days, 60days and 30days**

|  | **BMI quartile** | **Infection Present** | |  | **Infection Absent** | |  |
| --- | --- | --- | --- | --- | --- | --- | --- |
|  |  | **HR (95% CI)** | | ***P*** | **HR (95% CI)** | | ***P*** |
| **100 days** | Q1(≤20 kg/m^2^) | 1.82 | (1.51, 2.19) | <0.001 | 1.39 | (1.16, 1.67) | <0.001 |
|  | Q2(21–23 kg/m^2^) | 1.44 | (1.18, 1.75) | <0.001 | 1.16 | (0.96, 1.40) | 0.124 |
|  | Q3 (24–26 kg/m^2^) | 1.40 | (1.12, 1.74) | 0.003 | 1 | Ref |  |
|  | Q4 (≥27 kg/m^2^) | 1.06 | (0.84, 1.35) | 0.623 | 0.92 | (0.74, 1.14) | 0.426 |
|  |  |  |  |  |  |  |  |
|  | **BMI quartile** | **Infection Present** | |  | **Infection Absent** | |  |
|  |  | **HR (95% CI)** | | ***P*** | **HR (95% CI)** | | ***P*** |
| **60 days** | Q1(≤20 kg/m^2^) | 1.76 | (1.45, 2.13) | <0.001 | 1.39 | (1.16, 1.68) | <0.001 |
|  | Q2(21–23 kg/m^2^) | 1.42 | (1.16, 1.74) | 0.001 | 1.16 | (0.96, 1.41) | 0.127 |
|  | Q3 (24–26 kg/m^2^) | 1.39 | (1.10, 1.74) | 0.005 | 1 | Ref |  |
|  | Q4 (≥27 kg/m^2^) | 1.02 | (0.79, 1.31) | 0.873 | 0.92 | (0.74, 1.15) | 0.473 |
|  |  |  |  |  |  |  |  |
|  | **BMI quartile** | **Infection Present** | |  | **Infection Absent** | |  |
|  |  | **HR (95% CI)** | | ***P*** | **HR (95% CI)** | | ***P*** |
| **30 days** | Q1(≤20 kg/m^2^) | 1.74 | (1.42, 2.14) | <0.001 | 1.38 | (1.14, 1.69) | 0.001 |
|  | Q2(21–23 kg/m^2^) | 1.32 | (1.05, 1.64) | 0.016 | 1.15 | (0.94, 1.41) | 0.168 |
|  | Q3 (24–26 kg/m^2^) | 1.36 | (1.06, 1.74) | 0.016 | 1 | Ref |  |
|  | Q4 (≥27 kg/m^2^) | 0.94 | (0.71, 1.25) | 0.677 | 0.92 | (0.73, 1.16) | 0.458 |

Top; 100 day in hospital mortality, Middle; 60 day in hospital mortality, Bottom; 30 day in hospital mortality. Cox proportional hazards analysis adjusted for demographics, medical history: age, sex, hypertension, anemia, malignancy, reason for admission, history of ambulance transportation, history of ICU admission, history of vasopressor usage, history of blood transfusion, and history of usage of central venous line.

HR, hazard ratio; CI, confidence interval; BMI, body mass index; Ref, reference;
