# Supplementary material for: Short-term prognosis of emergently hospitalized dialysis-independent chronic kidney disease patients: A nationwide retrospective cohort study in Japan
Source: PLoS One. 2018 Nov 29;13(11):e0208258. doi: 10.1371/journal.pone.0208258 (PMC6264841; doi:10.1371/journal.pone.0208258)
Supplement: S3 Table — (DOCX) [file pone.0208258.s003.docx]

**S3 Table. Comparison of All-cause mortality by BMI and infection in DI-CKD patients *without* DM, within 100 days, 60days and 30days**

|  | **BMI quartile** | **Infection Present** | |  | **Infection Absent** | |  |
| --- | --- | --- | --- | --- | --- | --- | --- |
|  |  | **HR (95% CI)** | | ***P*** | **HR (95% CI)** | | ***P*** |
| **100 days** | Q1(≤20 kg/m^2^) | 1.62 | (1.31, 2.00) | <0.001 | 1.34 | (1.09, 1.66) | 0.006 |
|  | Q2(21–23 kg/m^2^) | 1.30 | (1.03, 1.63) | 0.027 | 1.12 | (0.90, 1.40) | 0.319 |
|  | Q3 (24–26 kg/m^2^) | 1.20 | (0.93, 1.57) | 0.167 | 1 | Ref |  |
|  | Q4 (≥27 kg/m^2^) | 0.93 | (0.70, 1.25) | 0.632 | 0.87 | (0.66, 1.14) | 0.299 |
|  |  |  |  |  |  |  |  |
|  | **BMI quartile** | **Infection Present** | |  | **Infection Absent** | |  |
|  |  | **HR (95% CI)** | | ***P*** | **HR (95% CI)** | | ***P*** |
| **60 days** | Q1(≤20 kg/m^2^) | 1.52 | (1.22, 1.89) | <0.001 | 1.30 | (1.05, 1.60) | 0.018 |
|  | Q2(21–23 kg/m^2^) | 1.26 | (1.00, 1.60) | 0.055 | 1.07 | (0.85, 1.34) | 0.577 |
|  | Q3 (24–26 kg/m^2^) | 1.24 | (0.94, 1.62) | 0.127 | 1 | Ref |  |
|  | Q4 (≥27 kg/m^2^) | 0.97 | (0.71, 1.32) | 0.830 | 0.88 | (0.67, 1.15) | 0.350 |
|  |  |  |  |  |  |  |  |
|  | **BMI quartile** | **Infection Present** | |  | **Infection Absent** | |  |
|  |  | **HR (95% CI)** | | ***P*** | **HR (95% CI)** | | ***P*** |
| **30 days** | Q1(≤20 kg/m^2^) | 1.53 | (1.21, 1.94) | <0.001 | 1.33 | (1.06, 1.67) | 0.015 |
|  | Q2(21–23 kg/m^2^) | 1.26 | (0.97, 1.63) | 0.079 | 1.08 | (0.85, 1.38) | 0.509 |
|  | Q3 (24–26 kg/m^2^) | 1.25 | (0.93, 1.68) | 0.136 | 1 | Ref |  |
|  | Q4 (≥27 kg/m^2^) | 0.88 | (0.62, 1.25) | 0.477 | 0.93 | (0.70, 1.23) | 0.600 |

Top; 100 day in hospital mortality, Middle; 60 day in hospital mortality, Bottom; 30 day in hospital mortality. Cox proportional hazards analysis adjusted for demographics, medical history: age, sex, hypertension, anemia, malignancy, reason for admission, history of ambulance transportation, history of ICU admission, history of vasopressor usage, history of blood transfusion, and history of usage of central venous line.

.

HR, hazard ratio; CI, confidence interval; BMI, body mass index; Ref, reference;
