# Supplementary material for: Short-term prognosis of emergently hospitalized dialysis-independent chronic kidney disease patients: A nationwide retrospective cohort study in Japan
Source: PLoS One. 2018 Nov 29;13(11):e0208258. doi: 10.1371/journal.pone.0208258 (PMC6264841; doi:10.1371/journal.pone.0208258)
Supplement: S4 Table — (DOCX) [file pone.0208258.s004.docx]

**S4 Table. Comparison of All-cause mortality by BMI and infection in DI-CKD patients *with* DM, within 100 days, 60days and 30days**

|  | **BMI quartile** | **Infection Present** | |  | **Infection Absent** | |  |
| --- | --- | --- | --- | --- | --- | --- | --- |
|  |  | **HR (95% CI)** | | ***P*** | **HR (95% CI)** | | ***P*** |
| **100 days** | Q1(≤20 kg/m^2^) | 2.80 | (1.92, 4.08) | <0.001 | 1.59 | (1.09, 2.32) | 0.016 |
|  | Q2(21–23 kg/m^2^) | 1.95 | (1.32, 2.89) | <0.001 | 1.40 | (0.98, 2.01) | 0.061 |
|  | Q3 (24–26 kg/m^2^) | 1.90 | (1.26, 2.87) | 0.003 | 1 | Ref |  |
|  | Q4 (≥27 kg/m^2^) | 1.17 | (0.76, 1.81) | 0.444 | 1.13 | (0.77, 1.64) | 0.532 |
|  |  |  |  |  |  |  |  |
|  | **BMI quartile** | **Infection Present** | |  | **Infection Absent** | |  |
|  |  | **HR (95% CI)** | | ***P*** | **HR (95% CI)** | | ***P*** |
| **60 days** | Q1(≤20 kg/m^2^) | 2.69 | (1.82, 3.97) | <0.001 | 1.60 | (1.09, 2.36) | 0.016 |
|  | Q2(21–23 kg/m^2^) | 1.90 | (1.27, 2.85) | 0.002 | 1.45 | (1.01, 2.09) | 0.048 |
|  | Q3 (24–26 kg/m^2^) | 1.85 | (1.21, 2.83) | 0.005 | 1 | Ref |  |
|  | Q4 (≥27 kg/m^2^) | 1.18 | (0.75, 1.84) | 0.474 | 1.09 | (0.74, 1.59) | 0.664 |
|  |  |  |  |  |  |  |  |
|  | **BMI quartile** | **Infection Present** | |  | **Infection Absent** | |  |
|  |  | **HR (95% CI)** | | ***P*** | **HR (95% CI)** | | ***P*** |
| **30 days** | Q1(≤20 kg/m^2^) | 2.60 | (1.72, 3.92) | <0.001 | 1.48 | (0.99, 2.21) | 0.058 |
|  | Q2(21–23 kg/m^2^) | 1.43 | (0.91, 2.24) | 0.122 | 1.36 | (0.93, 2.00) | 0.113 |
|  | Q3 (24–26 kg/m^2^) | 1.68 | (1.06, 2.66) | 0.027 | 1 | Ref |  |
|  | Q4 (≥27 kg/m^2^) | 1.08 | (0.67, 1.75) | 0.748 | 0.95 | (0.64, 1.42) | 0.800 |

Top; 100 day in hospital mortality, Middle; 60 day in hospital mortality, Bottom; 30 day in hospital mortality. Cox proportional hazards analysis adjusted for demographics, medical history: age, sex, hypertension, anemia, malignancy, reason for admission, history of ambulance transportation, history of ICU admission, history of vasopressor usage, history of blood transfusion, and history of usage of central venous line.

HR, hazard ratio; CI, confidence interval; BMI, body mass index; Ref, reference;
